# Effect of PCV-2 Vaccination on Cytokines Gene Expression Profile in Wild Boar Peripheral Blood Mononuclear Cells after Stimulation with Mycobacteria Antigens

**DOI:** 10.1155/2024/7308995

**Published:** 2024-04-13

**Authors:** Rocío Holgado-Martín, David Risco, Alfredo García-Sánchez, Remigio Martínez-Pérez, José Manuel Benítez-Medina, Alfonso Ramos, Javier Hermoso-De Mendoza, Luis Gómez

**Affiliations:** ^1^Departamento de Medicina Animal, Área de Anatomía Patológica y Anatomía Comparada, Facultad de Veterinaria de Cáceres, Universidad de Extremadura, Cáceres 10003, Spain; ^2^Centro de Investigación Científica y Tecnológica de Extremadura (CICYTEX) km 372 06187 Guadajira, Badajoz, Spain; ^3^Departamento de Sanidad Animal, Grupo de Investigación en Sanidad Animal y Zoonosis (GISAZ), UIC Zoonosis y Enfermedades Emergentes ENZOEM, Universidad de Córdoba (ROR code 05yc77b46), 14014, Córdoba, Spain; ^4^Departamento de Salud Animal, Unidad Patología Infecciosa, Facultad de Veterinaria de Cáceres, Universidad de Extremadura, Cáceres 10003, Spain; ^5^Departamento de Matemáticas, Unidad de Estadística e Investigación Operativa, Facultad de Veterinaria de Cáceres, Universidad de Extremadura, Cáceres 10003, Spain

## Abstract

Wild boar (*Sus scrofa*) is a common wild ungulate known as the most important reservoir of tuberculosis (TB) in Spain. The severity of TB lesions in this species and the high prevalence of porcine circovirus type 2 (PCV-2) have been related. PCV-2 is ubiquitous in swine populations, being usual for the free-living ones the contact with this agent. Recent studies found a correlation between a decrease of generalised TB prevalence in wild boar populations and the PCV-2-vaccination. The aim of this study was to find out if PCV-2 vaccination modulates the gene expression of cytokines from immune cells after its exposition with mycobacterial antigens using an in vitro methodology. A total of 46 wild boars from a PCV-2 infection endemic area were blood-sampled before and after the PCV-2 vaccination of 22 of them. Peripheral blood mononuclear cells (PBMC) were obtained and isolated from these samples. Aliquots of the cells were in vitro cultured and respectively stimulated with PPDa, PPDb, and a mitogen. A complete analysis of the gene expression of cytokines from the cultured PBMC was carried out. Also, *Mycobacterium bovis* and PCV-2 contacts were revealed by ELISA and/or qPCR. The results demonstrated that the animals which have had contact with PCV-2 and had been vaccinated, manifested a significant decrease in gene expression of proinflammatory cytokines, like interleukin 1 beta, interleukin 6, and tumour necrosis factor-alpha, possibly related with the severity of TB lesions, and also a significant decrease of interleukin 10, a key cytokine. In conclusion, in case of possible infection or contact events with the virus, PCV-2 vaccination could be an effective measure to reduce the TB severity in wild boar populations, which could decrease the intra and interspecies transmission of TB.

## 1. Introduction

Wild boar (*Sus scrofa*) is a wild ungulate abundant in some European countries like Spain. This species may act as a reservoir of several pathogens that cause diseases shared with domestic animals, like tuberculosis (TB) [1]. The main causative agent of this disease is *Mycobacterium tuberculosis* variant *bovis*, formerly called *M. bovis*, belonging to the *M. tuberculosis* complex (MTBC) [2], which produces a chronic condition, leading to worldwide significant economic losses in livestock, since it affects both animal productivity and herd demographic composition [3–5]. Wild boar has been pointed out as the main reservoir of this pathogen in south-central Spain, where high prevalence of infections have been usually found [6]. This fact has been related with the existence of positive cattle herds sharing habitat with wild boar, suggesting a key role in the maintenance of TB in cattle [7, 8].

The prevalence of TB and the severity of lesions displayed by affected wild boar have been positively correlated with the existence of coinfections with porcine circovirus type 2 (PCV-2) [9] and the presence of other concomitant pathogens like *Metastrongylus* spp. [10]. In fact, recent studies have shown that the implementation of measures focused on the control of these pathogens (single-dose vaccination against PCV-2 and repetitive deworming with ivermectin) could be useful to reduce the severity of TB lesions in wild boar. The animals inoculated with one dose of PCV-2 vaccine develop less severe tuberculous lesions, since it seems that the mycobacteria remain confined to head lymph nodes and do not spread to the rest of the organism [11–13].

PCV-2 is a ubiquitous agent causative of several clinical diseases, such as PCV-associated disease [14, 15]. PCV-2 persists in the host, causing a prolonged pro-inflammatory status and leading to immunopathological effects. This virus shows a strong tropism for lymphoid tissues [16, 17], causing a deep wasting of the immune system [18]. The most characteristic lesions associated with PCV-2 are lymphocyte depletion caused by the destruction of lymphoid-architecture and its replacement by histiocytic cells, besides to granulomatous inflammation [19–22]. These lesions may lead to severe immunosuppression in the affected animals, which favour the infection and development of chronic diseases such as TB or even its reactivation when the disease is in a latent stage [23]. PCV-2 infection could compromise key routes for the control of TB, such as the triggering of a Type 1 T-cells (Th1) response or macrophage activation, and these actions are mediated by different cytokines [12]. The Th1 response is characterised by the secretion of interferon-gamma (IFN-*γ*) and interleukin 12 (IL-12) cytokines, which have been demonstrated to be released by infection of *M. tuberculosis* [24]. Also, some studies found an association between the development of severe lesions of TB and the overproduction of proinflammatory cytokines, such as interleukin 1 beta (IL-1*β*), interleukin 6 (IL-6), and tumour necrosis factor-alpha (TNF-*α*), which trigger the inflammatory response [25–27]. Another relevant cytokine in the immune response is interleukin 10 (IL-10), an anti-inflammatory cytokine that is key to monocytes differentiation to M1 (phagocytic cells) or M2 macrophages (cell repair), favouring the differentiation towards this second pathway, which could relate the presence of this cytokine with reparation of lesional processes [25].

Despite the association between PCV-2 and TB severity, which has been reported in wild boars, the immunological mechanisms that modulate TB development in animals infected by PCV-2 or vaccinated against this virus have not been studied yet. The aim of this study was to explore the influence of PCV-2 vaccination on the relative expression of genes related with cytokines production in peripheral blood mononuclear cells (PBMCs) after exposition to *M. bovis* and *M. avium* antigens and pokeweed, a mitogen.

## 2. Materials and Methods

### 2.1. Experimental Design

The experiment was conducted in a fenced wild boar game estate located in the limit between Extremadura and Castilla La-Mancha regions, in middle-western of Spain (Lt: 39° 30' 12.80” N. Lg: 5° 11' 3.49” W). The fenced area has a typical continental Mediterranean climate, with hot, dry summers and mild and moderately wet winters. The vegetation is mainly composed by scrublands of *Cistus* spp. and *Arbutus unedo* and evergreen oak forests (*Quercus ilex* and *Quercus suber*). The fenced estate is used to hunt three different species of ungulates: wild boar, red deer (*Cervus elaphus*), and European mouflon (*Ovis orientalis musimon*). Wild boars are raised in a TB-free fence and tested by serology annually. When they are 1 year old, animals are released to a hunting fence. Management for collecting samples is carried out in stockyards with a trap, where each individual is completely immobilised. When the animals are captured for serologic analysis, they are identified with a microchip on the right side of the neck.

To carry out the experiment, 46 animals were captured for *in vivo* sampling. These animals were approximately 12 months old and were living in a specific fenced area of 2 hectares to avoid external contacts, being supplied with water and feed using artificial drinkers and feeders. Two different groups were formed: vaccinated (V) and non-vaccinated (NV). The vaccinated group was formed by 22 randomly chosen animals, which were blood sampled using EDTA tubes by puncturing the ophthalmic sinus and then parenterally inoculated (intramuscular) with a single dose of inactivated PCV-2 vaccine (CIRCOVAC®, Merial, France). The non-vaccinated group comprised the remaining 24 animals, which were likewise sampled but did not receive vaccination, being used as a control group. After sampling, all animals were immediately released to their fence. The vaccine-induced immunity should raise after 2–14 weeks, according to manufacturer information. One month after the vaccination of the V group, blood samples were taken again from both groups, so at the end of the experiment, we had samples of pre-vaccination and post-vaccination status. All sampled animals shared the same epidemiological scenario during the experiment.

### 2.2. TB and PCV-2 Diagnosis

Serological tests of both *M. bovis* and PCV-2 infections were carried out on the wild boar serum samples obtained at the beginning of the experience. A commercial indirect ELISA kit (INgezim TB porcine®, INGENASA, Gold Standard Diagnostics Madrid, Spain) was used to detect the presence of antibodies against *M. bovis*, following manufacturer instructions. Similarly, a commercial double Ig detection ELISA kit (ELISA kit INgezim Circovirus IgM/IgG INGENASA, Gold Standard Diagnostics Madrid, Spain) was used to assess the presence of antibodies against PCV-2, following manufacturer instructions.

Furthermore, a qPCR assay was carried out to detect PCV-2 DNA in serum samples obtained at the beginning and the final of the experience. PCR assays were conducted using RealQ Plus 2x Master Mix for Probe low ROXᵀᴹ (AMPLIQON, Denmark) after DNA extraction from serum with Nukex Mag DNA isolation (Gerbion GmbH & Co., Germany) according to manufacturer instructions, using a KingFisherᵀᴹ Flex Purification System (ThermoFisherᵀᴹ Scientific Inc., USA) automatic extractor.

### 2.3. PBMCs Extraction and *In Vitro* Stimulation

In this study, the lymph cells used for stimulation were PBMCs collected from pre- and post-vaccination blood samples of the same animal. The PBMC extraction was carried out immediately in the next 2 hr after blood collection. A modified protocol for the isolation of mononuclear cells and granulocytes from human blood was followed [28, 29]. Then, each sample was divided into four parts (125 *µ*L), each one stimulated with a different compound: PPDb tuberculin (purified protein derivative from cultures of *Mycobacterium bovis*), PPDa tuberculin (purified protein derivative from cultures of *Mycobacterium avium*), Pokeweed mitogen (ID.vet, France) (a lectin derived from the roots of *Phytolacca americana*, which is used to assess cellular immunocompetence) and phosphate-saline buffer (as control). The concentrations used of these stimulants are indicated in Table 1. After 24 hr of stimulation at 37°C, all samples were subjected to RNA extraction following manufacturer instructions (MagMAXᵀᴹ *mir*Vanaᵀᴹ Total RNA isolation kit, Applied Biosystems, USA) in an automatic extractor KingFisherᵀᴹ Flex Purification System (ThermoFisherᵀᴹ Scientific Inc., USA). All samples were analysed in order to know RNA concentration (ng/*µ*L), with a NanoDrop 2000 (ThermoFisher, USA) and stored at −80°C until use. Finally, eight RNA samples from each specimen of both groups (V and NV) were obtained: the four different stimulations from the pre-vaccination situation and the four different stimulations from the post-vaccination situation.

### 2.4. Gene Expression

Subsequently, RT-qPCR was carried out using TaqPathᵀᴹ 1-step Multiplex Master Mix (Applied Biosystems, USA). Nine genes of cytokines were analysed: IL-1*β*, interleukin 2 (IL-2), interleukin 4 (IL-4), IL-6, interleukin 8 (IL-8), IL-10, IL-12p40, IFN-*γ*, and TNF-*α*; GAPDH was used as reference gene. The genes evaluated and their primers and probes sequences are referenced in Table S1. The final concentrations in the RT-qPCR reaction of primers were 0.5 and 0.1 *µ*M of probe for all genes [30]. The volume of RNA analysed was 3 *µ*L. Results analysis was done by taking the Ct mean from each sample and applying the formula recommended to obtain the fold gene expression of the different cytokines *∆∆*Ct = (((*∆*Ct cytokine-stimulated sample − *∆*Ct reference gene stimulated sample) − (*∆*Ct cytokine non stimulated sample − *∆*Ct non-stimulated reference gene expression)) *x* −1)^2^ [31].

### 2.5. Statistical Analysis

From the results of gene expression of cytokines, three new variables were created, one for proinflammatory cytokines, where the mean folds of IL-1*β*, IL-6, and TNF-*α* were taken and named as proinflammatory (Proinf); the other two variables were created to evaluate the immune response via Th1 or Th2. Th1 variable included IL-12p40 and IFN-*γ* folds. Th2 variable included IL-4 and IL10 folds. Also, all results of the cytokines were analysed individually, so that the gene expression of each cytokine was statistically analysed.

*∆∆*Ct was used to carry out statistical analysis that was done with Rstudio software (4.3.1 version). Shapiro–Wilk test was used to check the normality, and the Wilcoxon test was used to reveal the significant differences between means. An analysis of *∆∆*Ct means obtained between V and NV groups was carried out with the total of animals on one side and with animals that had been in contact with PCV-2 (revealed by serology or PCR) on the other side.

## 3. Results

### 3.1. PCV-2 and TB Diagnosis

All analysed animals were negative to the ELISA TB test in both samplings (pre- and post-vaccination). An active infection of PCV-2 was observed in 5 animals from 46 (10.9%) analysed by ELISA and 7 out of 46 (15.2%) with less recent infection, resulting in a total of 12 animals with PCV-2 contact determined by serology (26.1%). Moreover, using PCV-2 qPCR analysis, only three positive animals (6.5%) were observed, two of them in the samples obtained before the vaccination.

### 3.2. Cytokine Gene Relative Expression

From 46 animals sampled, a total of 368 samples of stimulated PBMC were obtained, resulting from two samplings on each animal (pre- and post-vaccination) and from dividing each sample into four aliquots. There were no significant differences between means of NV and V animals for all cytokines neither before nor after treatment (see Table S2).

However, if only means of animals with a previous contact with PCV-2 (ELISA or PCR) were analysed (13 animals), a significant difference was observed between NV and V (see Table 2). IL-1*β* (6.62 vs. 0.77, *p*-value 0.03; W = 32), IL-6 (9.95 vs. 1.87, *p*-value 0.01; W = 34), and TNF-*α* (3.92 vs. 1.99, *p*-value 0.03; W = 32) cytokines expressions were significantly below in V group with mitogen stimulation comparing with NV. As a consequence, the variable called “Proinf” (20.50 vs. 4.63, *p*-value 0.02; W = 33), with IL-1*β*, IL-6, and TNF-*α* means, show significant differences in means between V and NV group, being the expression lower in V group. For IL-10 (4.47 vs. 1.66, *p*-value 0.03; W = 32) cytokine expression, a significant difference in means between NV and V in PPDb stimulation was observed, being lower in V group. Such significance was not observed for PPDa and mitogen stimulation for IL-10 expression. PPDa stimulation did not have significant differences in any case. The remaining cytokines studied did not have significant results in any instance.

## 4. Discussion

The results show that vaccination against PCV-2 in wild boar with previous contact with this virus, modulates the expression of different cytokines from PBMC when they are in vitro-exposed to different antigens, like PPDb.

PBMC from vaccinated wild boar showed a lower expression of the IL-10 synthesis codification gene than non-vaccinated animals when they were stimulated with PPDb in vitro. The IL-10 is a key cytokine that favours the differentiation of monocytes M2 macrophages (cell repair). Therefore, the under-expression of this cytokine favours an M1 differentiation of monocytes, resulting in macrophages, which are activated, having great phagocytic capacity, and being useful to fight against intracellular pathogens such as *M. bovis* [25]. For this cytokine, no significant differences in the mean fold increase were found after in vitro PBMC stimulations with PPDa and mitogen. Nevertheless, mitogen stimulations seem to have a similar trend like PPDb stimulations in order to under-express cytokines in the vaccinated group.

Also, when PBMC were exposed to mitogen, vaccinated animals showed an under-expression of coding genes for IL-1*β*, IL-6, and TNF-*α* compared with non-vaccinated animals. These three cytokines are included in the proinflammatory group, which functions are to trigger and maintain the inflammatory response [25, 32]. An under-expression of these cytokines indicates a reduction of inflammatory phenomena, oedema, neutrophils recruitment, etc. These processes can have pathological consequences when exacerbated [33]. Significant statistical differences were not observed regarding these cytokines from samples stimulated with PPDb, although the trend is similar to that observed in mitogen-stimulated samples (lower levels of IL-1*β*, IL-6, and TNF-*α* expression in vaccinated animals). In this sense, the development of severe lesions of TB has been related to an excessive production of these proinflammatory cytokines, with the greater attraction of neutrophils, that aggravate the injury set [25–27]. In fact, it has been proven in humans that specific treatments conducted to reduce this inflammatory response using AINES, like Ibuprofen, have given promising results [34].

The gene expression on samples stimulated with PPDa antigens did not show statistical differences between PCV-2 vaccinated and non-vaccinated animals. Also, the cytokines IL-2 and IL-8 had no statistical differences in any type of stimulation, despite its role in inflammatory response [25].

The possibility of any animal to have had previous contact with *M. bovis* or *M. avium* could have altered the gene expression results of PBMC. Nevertheless, all animals included in this experiment were seronegative to *M. bovis*, suggesting the absence of previous contacts with this agent. However, the existence of previous or recent infection by PCV-2 was detected by ELISA or PCR in a total of 13 wild boars (nine vaccinated and four non-vaccinated). Indeed, when analysing only these animals with PCV-2 contact, significant differences in the gene expression of these cytokines (IL-1*β*, IL-6, IL-10, and TNF-*α*) were observed after comparing vaccinated and non-vaccinated animals. Several studies verify that PCV-2 antigen stimulation acts by up-regulating the expression of IFN-*γ*, IL-10, and proinflammatory cytokines, such as IL-1*β* and TNF-*α*, and scarce production of IL-4 and IL-2 is observed in PCV-2 infected pigs [33, 35, 36]. Thus, reducing the effect of PCV-2 infections through vaccination could modulate the over-expression of these cytokines in lymphoid cells from infected animals, as we have observed in this in vitro experiment [12].

The lower expression of IL-1*β*, IL-6, IL-10, and TNF-*α* in PCV-2 vaccinated animals could be related with the manifestation of less severe tuberculous lesions in animals co-infected with MTBC and PCV-2. This virus is relatively ubiquitous, and it is easy for free-living animals to result infected. So, PCV-2 vaccination could be an effective measure to reduce the TB transmission and severity of disease in wild boar populations, as a result of the reduction in the gene expression of proinflammatory cytokines. Nevertheless, there have not been significant differences for the relative expression of other cytokines, which are very important for immune response against MTBC, like IFN-*γ* and IL-12p40. These cytokines favour the development of acquired Th1 immune response or macrophage activation. These actions are fundamental to fight against MTBC infections in an efficient way [24, 27]. Also, the under-expression of IL-10, considered an anti-inflammatory cytokine, could be contrary to the expected results of the under-expression of proinflammatory cytokines, so more inquiry is required about this issue. Therefore, further studies will be necessary in order to continue deciphering the immunological interactions between PCV-2 and TB in wild boar.

## 5. Conclusions

The present *in vitro* study shows that PCV-2 vaccination modulates the gene expression of cytokines in wild boars that have had previous contact with the virus, when they are exposed to MTBC antigens. PPDb *in vitro* stimulation of PBMC in PCV-2 vaccinated wild boar, reduces IL-10 expression and could favour the phagocytic activity. The *in vitro* mitogen stimulation of PBMC in PCV-2 vaccinated wild boar, reduce the IL-1*β*, IL-6, and TNF-*α*, which could decrease the inflammatory reaction since they are proinflammatory cytokines.

## Figures and Tables

**Table 1:** List of stimulants, quantity, and concentrations for each sample.

Stimulants	*µ*L for 1 sample	Total *µ*L	mg/mL
PPDb + PSB	2.75 + 9.75	12.5	0.0275
PPDa + PSB	5.5 + 7	12.5	0.0275
Mitogen + PSB	1.1 + 48.9	50	0.0275
PSB	50	50	0.4

**Table 2:** ELISA or PCR PCV-2 positive animals *∆∆*Ct means in the function of cytokine (IL-1*β*, IL-2, Il-4, IL-6, IL-8, IL-10, IL-12p40, IFN-*γ*, and TNF-*α*) or groups of cytokines (proinf, TH1, and TH2), group (NV/V) and type of stimulation (*M. bovis*, *M. avium*, and mitogen).

	IL-1*β*		IL-2		IL-4		IL-6	
	NV	V	NV	V	NV	V	NV	V
*M. bovis*	39.82	2.20	1.62	0.91	1.43	0.99	5.00	0.94
*M. avium*	2.75	2.76	1.79	0.88	0.91	0.89	1.90	0.98
Mitogen	6.62	**0.77 ^*∗∗*^**	1,319.84	150.11	73.26	21.56	9.95	**1.87 ^*∗∗*^**

	IL-8		IL-10		IL-12p40		IFN-*γ*	
	NV	V	NV	V	NV	V	NV	V

*M. bovis*	3.79	1.02	4.47	**1.66 ^*∗∗*^**	2.87	0.72	2.12	0.92
*M. avium*	1.75	1.58	1.51	1.31	17,216.03	2.25	1.35	0.63
Mitogen	3.22	1.34	6.58	1.68	5.98	2.80	17.52	4.92

	TNF-*α*		PROINF		TH1		TH2	
	NV	V	NV	V	NV	V	NV	V

*M. bovis*	4.91	1.67	49.73	4.81	6.61	2.59	5.55	2.65
*M. avium*	1.14	1.07	5.80	4.81	17,219.69	3.77	2.26	1.78
Mitogen	3.92	**1.99 ^*∗∗*^**	20.50	**4.63 ^*∗∗*^**	1,343.34	147.31	77.82	23.24

Statistical significance differences between groups are represented with  ^*∗∗*^ (*p*-value < 0.05). Bold values (V) have statistical significant differences with NV.

## Data Availability

The datasets generated and/or analysed during the study are available from the corresponding author upon reasonable request.

## Supplementary Materials

Supplementary Materials Table S1: sequences (5′−3′) of the primers and probes used for the detection of mRNA specific for cytokines and housekeeping genes by real-time RT-PCR in wild boar monocytes. Table S2: *∆∆*ct means of animals post-treatment in the function of cytokine or groups of cytokines, groups, and type of stimulation.
