# Supplementary material for: Effect of PCV-2 Vaccination on Cytokines Gene Expression Profile in Wild Boar Peripheral Blood Mononuclear Cells after Stimulation with Mycobacteria Antigens
Source: Transbound Emerg Dis. 2024 Apr 13;2024:7308995. doi: 10.1155/2024/7308995 (PMC12017140; doi:10.1155/2024/7308995)
Supplement: Supplementary Materials — Table S1: sequences (5′−3′) of the primers and probes used for the detection of mRNA specific for cytokines and housekeeping genes by real-time RT-PCR in wild boar monocytes. Table S2: ∆∆ct means of animals post-treatment in the function of cytokine or groups of cytokines, groups, and type of stimulation. [file 7308995.f1.docx]

Supplementary Materials

| **Gene-name** | **Sequence Fordward primer (sense)** | **Sequence Reverse primer (antisense)** | **Probe** |
| --- | --- | --- | --- |
| **IL-1β** | GTGCTGGCTGGCCCACA | GAACACCACTTCTCTCTTCA | FAM-CTCTCCACCTCCTCAAAGGG-BHQ1 |
| **IL-2** | TGCTGATCTCTCCAGGATGC | CCTCCAGAGCTTTGAGTTCTTCTACTA | FAM-AAGCAGGCTACAGAATTGAAACACCTT-BHQ1 |
| **IL-4** | GTCTGCTTACTGGCATGTACCA | GCTCCATGCACGAGTTCTTTCT | FAM-CCACGGACACAAGTGCGACATCACCTTAC-BHQ1 |
| **IL-6** | CTGGCAGAAAACAACCTGAACC | TGATTCTCATCAAGCAGGTCTCC | FAM-TGGCAGAAAAAGACGGATGC-BHQ1 |
| **IL-8** | AAGCTTGTCAATGGAAAAGAG | CTGTTGTTGTTGCTTCTCAG | FAM-TCTGCCTGGACCCCAAGGAAAAGT-BHQ1 |
| **IL-10** | CGGCGCTGTCATCAATTTCTG | CCCCTCTCTTGGAGCTTGCTA | FAM-AGGCACTCTTCACCTCCTCCACGGC-BHQ1 |
| **IL-12p40** | TCTTGGGAGGGTCTGGTTTG | AAGCTGTTCACAAGCTCAAGTATGA | FAM-ACCAGCAGCTTCTTCATCAGGGACATCA-BHQ1 |
| **IFN-γ** | CGATCCTAAAGGACTATTTTAATGCAA | TTTTGTCACTCTCCTCTTTCCAAT | FAM-ACCTCAGATGTACCTAATGGTGGACCTCTT-BHQ1 |
| **TNF-α** | AACCTCAGATAAGCCCGTCG | ACCACCAGCTGGTTGTCTTT | FAM-CCAATGCCCTCCTGGCCAACG-BHQ1 |
| **GAPDH** | ACATGGCCTCCAAGGAGTAAGA | GATCGAGTTGGGGCTGTGACT | FAM-CCACCAACCCCAGCAAGAGCACGC-BHQ1 |

**Supplementary table 1.** Sequences (5’-3’) of the primers and probes used for the detection of mRNA specific for cytokines and housekeeping genes by real-time RT-PCR in wild boar monocytes. IL=Interleukin; IFN=Interferon; TNF=Tumor necrosis factor; GAPDH=Glyceraldehyde-3-phosphate dehydrogenase (used like housekeeping). 5’-3’ modifications for the probes were as followed: FAM=6-carboxyfluorescein; BHQ=Black-Hole-Quencher.

|  | **IL-1β** | | **IL-2** | | **IL-4** | | **IL-6** | |
| --- | --- | --- | --- | --- | --- | --- | --- | --- |
|  | NV | V | NV | V | NV | V | NV | V |
| ***M. bovis*** | 8.73 | 2.15 | 1.12 | 1.45 | 1.3 | 0.73 | 1.7 | 1.31 |
| ***M. avium*** | 3.26 | 4.26 | 1.04 | 1 | 0.92 | 0.65 | 1.74 | 1.03 |
| **Mitogen** | 2.41 | 1.63 | 337.59 | 88.98 | 30.14 | 18.44 | 3.47 | 1.96 |
|  | **IL-8** | | **IL-10** | | **IL-12p40** | | **IFN-γ** | |
|  | NV | V | NV | V | NV | V | NV | V |
| ***M. bovis*** | 1.54 | 1.06 | 1.66 | 1.34 | 1.8 | 39.2 | 1.12 | 0.85 |
| ***M. avium*** | 1.24 | 1.51 | 1.03 | 1.23 | 6376644 | 12 | 1.06 | 0.95 |
| **Mitogen** | 1.51 | 1.33 | 2.14 | 1.8 | 1.72 | 111.87 | 5.25 | 4.4 |
|  | **TNF-α** | | **PROINF** | | **TH1** | | **TH2** | |
|  | NV | V | NV | V | NV | V | NV | V |
| ***M. bovis*** | 1.65 | 1.35 | 12.08 | 4.81 | 3.58 | 6.28 | 2.79 | 2.08 |
| ***M. avium*** | 1.33 | 1.49 | 6.32 | 6.79 | 6376646 | 14.04 | 1.91 | 1.72 |
| **Mitogen** | 1.67 | 1.85 | 7.55 | 5.45 | 357.92 | 198.3 | 31.83 | 20.24 |

**Supplementary table 2**. ∆∆ct means of animals post-treatment in function of cytokine (IL-1β, IL-2, Il-4, IL-6, IL-8, IL-10, IL-12p40, IFNγ and TNFα) or groups of cytokines (proinf, TH1 and TH2), groups (NV/V) and type of stimulation (*M. bovis*, *M.avium* and mitogen) (Pvalue<0,05).
